# Identification of lipopeptides in *Bacillus megaterium* by two-step ultrafiltration and LC–ESI–MS/MS

**DOI:** 10.1186/s13568-016-0252-6

**Published:** 2016-09-17

**Authors:** Yunxiao Ma, Qing Kong, Chong Qin, Yulin Chen, Yujie Chen, Ruihuan Lv, Guanghui Zhou

**Affiliations:** 1 School of Food Science and Engineering, Ocean University of China, Yushan Road 5, 62 Building, Qingdao, 266003 Shandong China; 2 Department of Medicinal Chemistry, College of Pharmacy, University of Michigan, Ann Arbor, MI USA

**Keywords:** Lipopeptide, Ultrafiltration, *Bacillus megaterium*, LC–ESI–MS, LC–ESI–MS/MS, Identification

## Abstract

A two-step ultrafiltration and liquid chromatography-electronic spray ionization-tandem mass spectrometry (LC–ESI–MS/MS) were applied to investigate the nature of lipopeptides in the methanol extract harvested from marine *Bacillus megaterium.* The structure of lipopeptide homologs were characterized by using collision-induced dissociation mass spectrometry analysis. Collectively, two kinds of linear fengycin A and B with a double bond were characterized for the first time besides the popular cyclic fengycin A and B with the difference in the amino acid at position 6 of the peptide moiety. It is worth noting that two kinds of linear isoforms of surfactins and two kinds of cyclic isoforms of esperin with a smaller peptide ring formed by the fifth l-Asp and *β*-hydroxy fatty acid were also separated and identified in addition to the all seven kinds of cyclic isoforms of surfactins. Only one analogue (bacillomycin D) of iturin family was identified in this research. To our best knowledge, this is the first report that more than 40 variants of lipopeptides from one strain of *Bacillus* were identified using our potent purification and identification methods.

**Electronic supplementary material:**

The online version of this article (doi:10.1186/s13568-016-0252-6) contains supplementary material, which is available to authorized users.

## Introduction

**Fig. 1:**
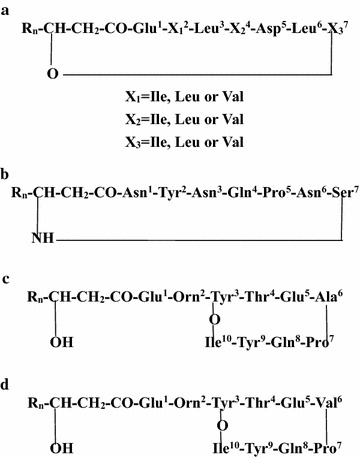
Basic structures of representative members and diversity within the three lipopeptide families. **a** Basic structures of surfactin. **b** Basic structures of iturin. **c** Basic structures of fengycin A. **d** Basic structures of fengycin B

Lipopeptides produced by members of *Bacillus* as secondary metabolites are amphiphilic molecules with hydrophilic and hydrophobic moieties (Ongena and Jacques 2008; Georgiou et al. 1992). Surfactins, iturins and fengycins are the three most well-known families of lipopeptides (Fig. 1). Surfactin is composed of an amphipathic, cyclic heptapeptide head group which is interlinked with a hydrophobic β-hydroxy fatty acid tail, comprising 12–16 carbon atoms (Hoefler et al. 2012; Rosenberg et al. 2016).The iturin family, represented by iturin A, C, D, E, bacillomycin D, F, L, Lc and mycosubtilin, are heptapeptides cyclized by a *β*-amino fatty acid chain containing 14–17 carbons (Romero et al. 2007; Yang et al. 2015). Members of the fengycin family, represented by fengycin A and B are decapeptides with an internal lactone ring in the peptidic moiety and with a *β*-hydroxy fatty acid with a length of 14–18 carbons that can be saturated or unsaturated (Ongena and Jacques 2008). Different strains of bacilli produce diverse types of cyclic lipopeptides. Related studies have shown that, some of the strains can produce only one family of lipopeptides while some strains can produce two or all three families of lipopeptides (Romero et al. 2007; Yang et al. 2015; Pecci et al. 2010). There is an increasing interest in lipopeptides because of their relatively nontoxic, biodegradable and unique structures which are suitable for their potential applications to many aspects of industry, ranging from biotechnology to environmental cleanup (Jacques 2011). The complexity and high cost of purification is the most important limitation for the commercial use of lipopeptides.

Ultrafiltration is highly suitable for the purification of lipopeptides from a series of extremely complex extract of bacteria and impurity. If the material concentration is higher than critical micelle concentration (CMC), the lipopeptide monomers will gather to form micelles with 2–3 times of molecular mass than monomers. But if methanol was added to micellar solution, micelles will recover into monomers. This feature can be used to process a two-step ultrafiltration for the purification of lipopeptides by choosing appropriate aperture of ultrafiltration membrane (Isa et al. 2007). Electrospray ionisation (ESI) tandem mass spectrometry was used to identify lipopeptides homologs produced by *B. licheniformis* (Pecci et al. 2010) and *B. subtilis* (Iatsenko et al. 2014). Surfactins, iturins and fengycins are all in the *m/z* range 1000–2000 that can be detected by ESI–MS (Yang et al. 2015; Sivapathasekaran et al. 2009).

A strain of marine *B. megaterium* CGMCC7086 was previously isolated by our group from the intestine of marine fish (Kong et al. 2010). In this work, lipopeptides were isolated and preliminarily purified from marine *B. megaterium* through a two-step ultrafiltration. A liquid chromatography-electronic spray ionization-tandem mass spectrometry (LC–ESI–MS/MS) method was also developed to characterize the structure and composition of lipopeptide families. Collision-induced dissociation (CID) mass spectrometry analysis was used to characterize the structure of lipopeptide homologs. Furthermore, over 40 lipopeptides homologs including bacillomycin D, surfactins, esperins and fengycins were identified. Of which, two broad types including surfactins and fengycins are linear lipopeptides which were seldom been reported. To our best knowledge, this is the first report that more than 40 variants of lipopeptides were identified in a single microorganism. The purification method and identification results can provide basis for large-scale preparation and widely application of lipopeptides.

## Materials and methods

### Bacterial strain and culture conditions

The marine *B. megaterium* strain CGMCC7086 used in this work was previously isolated from the intestines of marine fish. For production of the lipopeptides, an inoculum of marine *B. megaterium* into 50 mL of Luria–Bertani medium in 250-mL Erlenmeyer flask and incubated at 37 °C for 16 h with shaking at 180 rpm. Five percent of the 16-h-old inoculum was seeded into a 500-mL flask containing 200 mL fermentation medium (1 L: 20 g glucose, 1 g yeast extract, 7 g NH_4_NO_3_, 7 g NaCl, 0.78 g MgSO_4_·7H_2_O, 0.5 g KCl, 1.5 g KH_2_PO_4_, 0.0004 g CuSO_4_·7H_2_O, 0.005 g MnSO_4_·H_2_O, 0.0002 g FeSO_4_·7H_2_O, pH 7.0), and the culture was incubated at the same condition for 72 h.

### Extraction and purification of lipopeptides

One thousand milliliters of the 72-h-old fermentation broth of *B. megaterium* was centrifuged at 9000×*g* at 4 °C for 20 min (Hitachi, Tokyo, Japan). The supernatant fluid was collected and its pH was acidified to 2.0 with 6 M HCl and then incubated overnight at 4 °C for precipitating lipopeptides.

The precipitates were harvested by centrifuging again at 10,000×*g* at 4 °C for 20 min. And then, the precipitates were extracted twice with anhydrous methanol for 5 h and the insoluble impurities were removed by centrifugation at 8000×*g* at 4 °C for 20 min. The methanol was evaporated using a vacuum rotary evaporator at 50 °C to concentrate the lipopeptides and the resulting sample was subsequently dissolved in a certain volume of Milli-Q water.

The lipopeptides were preliminarily purified through two-step ultrafiltration by a Millipore tubular polysulfone ultrafiltration separation unit with membranes cut-off molecules smaller than 30,000 Da. First step, ultrafiltration membrane separation unit was used to deal with lipopeptides aqueous solutions and deep indentation fluid was collected. Then, the pH of the mixture was adjusted to 7.0 using 1.0 M NaOH and added a certain volume of methanol with slow stirring for 2 h. Second step, the same method was used and the upper filtration liquid was collected. The upper filtration liquid was further concentrated using vacuum evaporator and subsequently using vacuum freeze drying to obtain powdery production of lipopeptides for further purification. The lipopeptides mixture was dissolved in Milli-Q water and filtered through a 0.2 μm membrane filter (PALL Gelman Laboratory, New York, USA) to obtain a 500 μg/ml stock solution for mass spectra analysis.

### Liquid chromatography electrospray ionization mass spectrometry (LC–ESI–MS) analysis of lipopeptides

The separation of the lipopeptide factions and all mass spectrometry analyses were performed on a surveyor HPLC on line with a LCQ Fleet Ion Trap mass spectrometer (Thermo Fisher Scientific, Waltham, MA, USA) equipped with an electrospray ion source in positive ion mode. LC–ESI–MS spectra were measured by passing the analysis through a Zorbax SB-C_18_ column (Agilent, Santa Clara, USA) (2.1 × 100 mm, 3.5 μm particle size).

Lipopeptides were eluted by a component solvent system that solvent A was acetonitrile and solvent B was water, both injected with 0.1 % formic acid for facilitating protonation of the basic nitrogen compounds yielding protonated molecules [M + H]^+^. Two different elution programs were used with a flow rate of 200 μL/min and the column temperature was stayed at 35 °C. A 100 μL aliquot was injected into system and gradient strategy for surfactin was as follows: 0–3.5 min, 60 % A to 93 % A; 3.5–20 min, 93 % A and 7 % B. The gradient strategy for both iturin and fengycin were as follows: 0–9 min, 45 % A to 55 % A; 9–20 min, 55 % A and 45 % B. The electrospray source was operated at a spray voltage of 4 kV, a capillary voltage of 35 V, and a capillary temperature of 325 °C; simultaneously, LC–MS full scan positive modes were performed from in the range from *m*/*z* 200 to 2000.

ESI–MS/MS coupled with CID and the collision gas of helium were used for further identification of the amino acid sequence. The selected precursor ions were acquired in an auto LC–ESI–MS/MS modalities and then the data were analyzed by Xcalibur 2.1 (Thermo Fisher Scientific Inc, Waltham, MA, USA).

## Results

### LC–ESI–MS analysis of lipopeptide extract

**Fig. 2:**
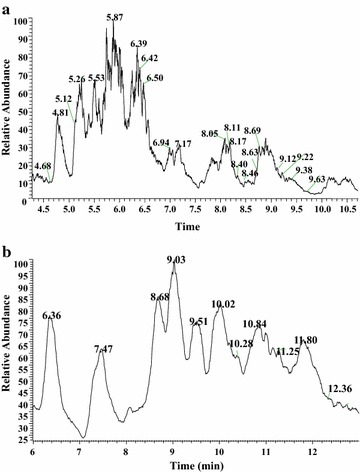
LC–ESI/MS analysis of the crude methanolic extract from the marine *B. megaterium* strain. **a** Chromatograms of the surfactin and its analogues, positive ion mode, TIC. **b** Chromatograms of the iturin and fengycin family, positive ion mode, TIC

**Fig. 3:**
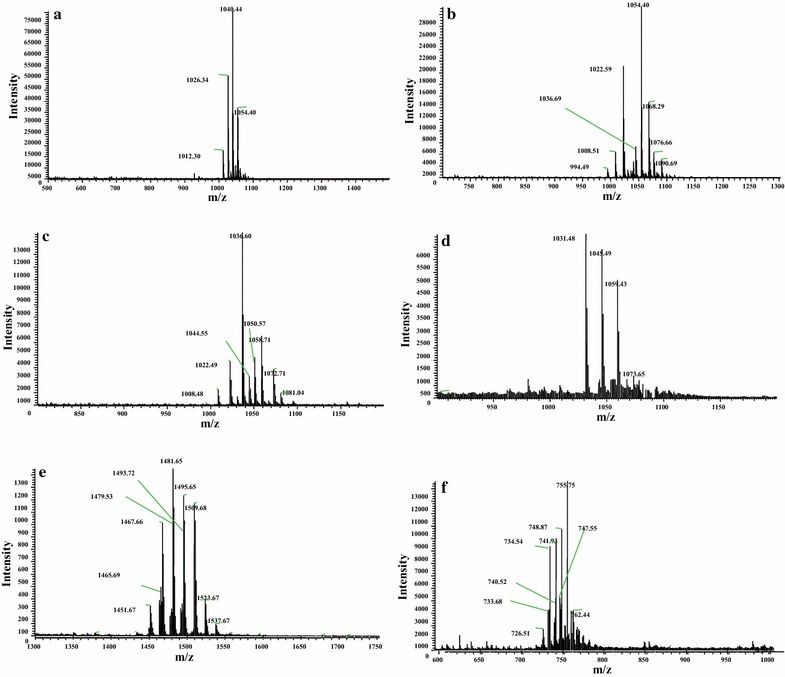
LC-ESI–MS lipopeptides spectrum. **a**–**c** Clusters of surfactin family. **d** Clusters of iturin family. **e** Clusters of fengycin family with single-charge molecule. **f** Clusters of fengycin family with double-charge molecular

To elute all putative lipopeptides, we used the designed elution program for surfactins and iturins as well as fengycins. The full scan LC–ESI–MS chromatogram for surfactins (Fig. 2a) showed the main peaks from 4 to 11 min. In the range 4–11 min, the main peaks of surfactin and its analogues were eluted at retention time (tR) 4.68, 4.81, 5.12, 5.26, 5.53, 5.87, 6.39, 6.42, 6.50, 6.94, 7.17, 8.05, 8.11, 8.17, 8.40, 8.46, 8.63, 8.69, 9.12, 9.22, 9.38 and 9.63 min, consistenting to molecules [M + H]^+^*m/z* 1050, 1012, 1050, 1026, 1026, 1040, 1054 (994), 994, 994, 1008 (1008, 1008, 1008), 1068, 1022, 1022, 1022, 1008 (1008), 1036, 1008, 1036, 1036 (1022), 1022, 1050 (1022) and 1050, as well as molecules [M + Na]^+^*m/z* 1072, 1034, 1072, 1048, 1048, 1062, 1076 (1016), 1016, 1016, 1030 (1030, 1030, 1030), 1090, 1044, 1044, 1044, 1030 (1030), 1058, 1030, 1058, 1058 (1044), 1044, 1072 (1044) and 1072 in positive modality. These data were shown in Fig. 3a–c.

The chromatogram for iturin and fengycin family (Fig. 2b) showed the main peaks from 6 to 13 min. In the range 6–13 min, the main peaks of a series of lipopeptides were eluted at retention time 6.36, 7.47, 8.68, 9.03, 9.51, 10.02, 10.28, 10.84, 11.25, 11.80 and 12.36 min, obeying to protonated molecules [M + H]^+^*m/z* 1031, 1045, 1467, 1481 (1059), 1495, 1509 (1073), 1451, 1465, 1479 (1479) 1493 (1493) and 1495 (1495), and also corresponding to sodiated molecules [M + Na]^+^*m/z* 1053, 1067, 1489, 1503 (1081), 1517, 1531 (1095), 1473, 1487, 1501 (1501) and 1515 (1505). It is noteworthy that the retention time at 8.68, 9.03, 9.51, 10.02, 10.28, 10.84, 11.25 and 11.80 min, they were revealed corresponding to protonated molecules [M + 2H]^2+^*m/z* 734, 741, 748, 755, 726, 733, 740 and 747. These results were shown in Fig. 3d–f. Some other [M + H]^+^ were 1523, 1537 and [M + 2H]^2+^ were 717 and 762.

### LC–ESI–MS/MS characterization of surfactin lipopeptides

**Fig. 4:**
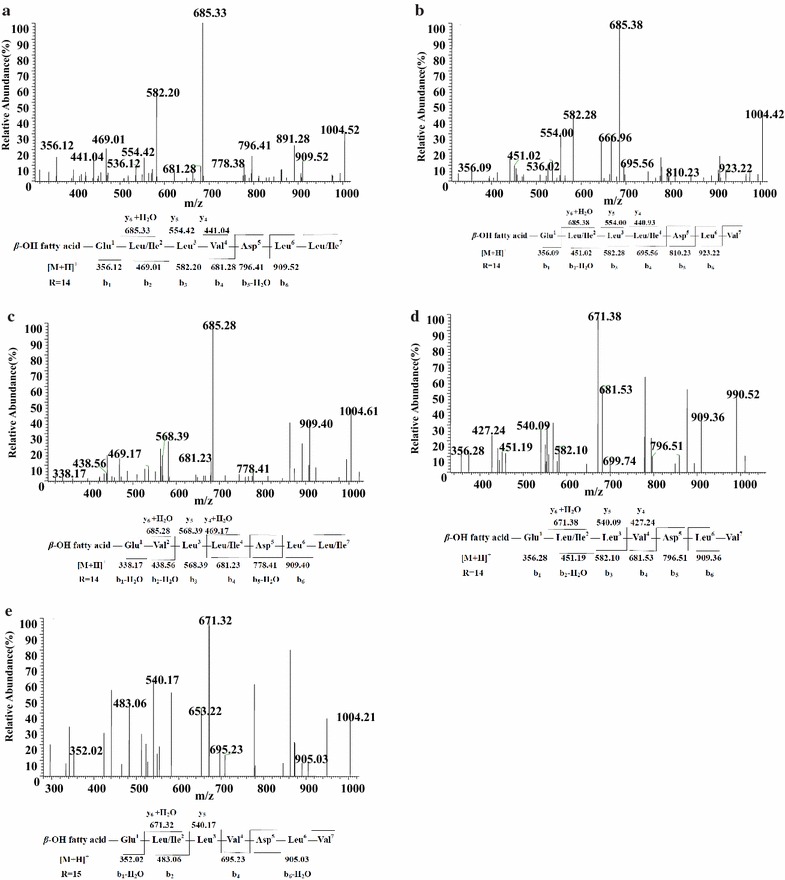
LC-ESI–MS/MS spectrum of the surfactin precursors. **a**–**c** Surfactin precursors ion [M + H]^+^ at *m/z* 1022 at retention time 8.05, 8.17, 8.11 min, respectively, containing a C14 *β*-hydroxy fatty acid chain. **d** Surfactin precursors ion [M + H]^+^ at *m/z* 1008 at retention time 8.40 min containing a Glu1-Leu/Ile2-Leu3-Val4-Asp5-Leu6-Val7 peptide and a C14 *β*-hydroxy fatty acid chain. **e** Surfactin precursors ion [M + H]^+^ at *m/z* 1022 at retention time 9.35 min containing a C15 *β*-hydroxy fatty acid chain

Figure 4a displayed the LC–ESI–MS/MS spectrum of the precursor ion at *m/z* 1022 with appearance of fragment ions at retention time 8.05 min, series of b^+^ and y^+^ ions could be easily assigned, which meaned the initial cleavage of protonated ester bond. The series of b^+^ fragment ions at *m/z* 1022(−H_2_O, 1004) → 909 → 796 → 681 → 582 → 469 → 356 were consistented with the losses of Leu/Ile^7^-Leu^6^-Asp^5^-Val^4^-Leu^3^-Leu/Ile^2^ from the C terminus. Furthermore, the second typical set of y^+^ fragment ions contained the peptidic moiety inside the C-terminal product ions at *m/z* 1022(−H_2_O, 1004) → 667(+H_2_O, 685) → 554 → 441 revealed the losses of C_14_*β*-hydroxyl fatty acid chain-Glu^1^-Leu/Ile^2^-Leu^3^ from the precursor ion. According to these typical CID fragments, the sequence could be deduced as C_14_*β*-hydroxyl fatty acid chain-Glu^1^-Leu/Ile^2^-Leu^3^-Val^4^-Asp^5^-Leu^6^-Leu/Ile^7^. Additionally, the LC–ESI–MS/MS spectra of protonated ions found at *m/z* 994 at retention time 6.50 min (Additional file 1: Figure S1a), 1008 at retention time 6.98 min (Additional file 1: Figure S1b), 1036 at retention time 9.12 min (Additional file 1: Figure S1c) and 1050 at retention time 9.38 min (Additional file 1: Figure S1d) with multiples of 14 Da (−CH_2_) difference, were proved to be homologs possessing the same amino acids sequence but different C_12_, C_13_, C_15_ and C_16_*β*-OH fatty acids, respectively. These data were in accordance with mass spectra of Sigma surfactin standard (S3523, Sigma-Aldrich, St. Louis, MO, USA) (data not shown).

The second peptide sequence was slightly different from the typical surfactins (Fig. 4b; Additional file 1: Figure S2). Figure 4b showed the LC–ESI–MS/MS spectrum of species observed as protonated ion at *m/z* 1022 at retention time 8.17 min. In the same way, it was suggested that the precursor ion possessed a peptide sequence of Glu^1^-Leu/Ile^2^-Leu^3^-Leu/Ile^4^-Asp^5^-Leu^6^-Val^7^ and a C_14_*β*-hydroxyl fatty acid chain. In addition, the LC–ESI–MS/MS spectra of protonated ions found at *m/z* 994 at retention time 6.38 min (Additional file 1: Figure S2a), 1008 at retention time 6.94 min (Additional file 1: Figure S2b), 1036 at retention time 8.63 min (Additional file 1: Figure S2c) and 1050 at retention time 9.63 min (Additional file 1: Figure S2d) with multiples of 14 Da (−CH2) difference, were proved to be homologs with the same amino acid sequence but different C_12_, C_13_, C_15_ and C_16_*β*-OH fatty acids.

Figure 4c and Additional file 1: Figure S3 showed a third surfactin isoforms of a series of homologs. Figure 4c showed the LC–ESI–MS/MS spectrum of species observed as protonated ion at *m/z* 1022 at retention time 8.11 min. Which was suggested the precursor ion possessed a peptide sequence of Glu^1^-Val^2^-Leu^3^-Leu/Ile^4^-Asp^5^-Leu^6^-Leu/Ile^7^ and a C_14_*β*-hydroxyl fatty acid chain. Simultaneously, the LC–ESI–MS/MS spectra of protonated ions found at *m/z* 994 at retention time 6.42 min (Additional file 1: Figure S3a), 1008 at retention time 6.89 min (Additional file 1: Figure S3b), 1036 at retention time 8.69 min (Additional file 1: Figure S3c) and with multiples of 14 Da (−CH_2_) difference, were proved to be homologs with the same amino acid sequence but different C_12_, C_13_ and C_15_*β*-OH fatty acids.

The fourth surfactin isoforms of LC–ESI–MS/MS spectrum of species observed as protonated ion were showed in Fig. 4d, e. Figure 4d showed fragment ions of precursor ion *m/z* 1008 at retention time 8.40 min. Then the sequence could be concluded based on the fragmentation profile as C_14_*β*-OH fatty acid—Glu^1^-Leu/Ile^2^-Leu^3^-Val^4^-Asp^5^-Leu^6^-Val^7^. In addition to this, a protonated ion *m/z* at 1022 at retention time 9.35 min was assigned as homologs with C_15_*β*-OH fatty acid (Fig. 4e).

**Fig. 5:**
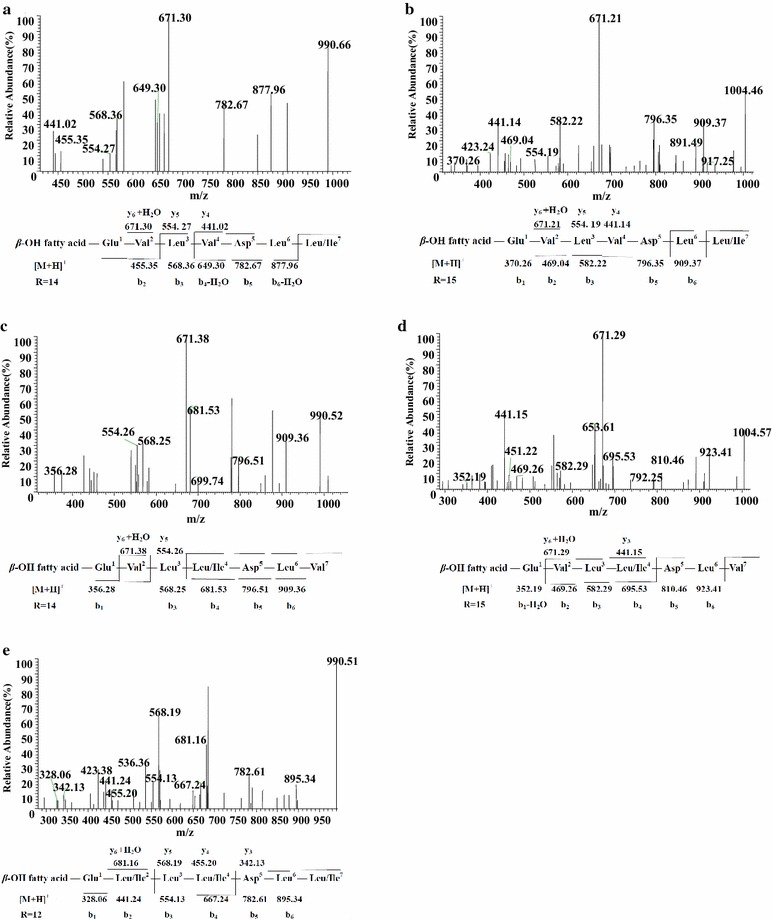
LC-ESI–MS/MS spectrum of the surfactin precursors. **a**, **b** Surfactin precursors ion [M + H]^+^ at *m/z* 1008 at retention time 8.46 and 9.22 min containing a Glu1-Val2-Leu3-Val4-Asp5-Leu6-Leu/Ile7 peptide and a C14 and C15 *β*-hydroxy fatty acid chain, respectively. **c**, **d** Surfactin precursors ion [M + H]^+^ at *m/z* 1008 at retention time 8.40 and 9.16 min containing a Glu1-Val2-Leu3-Leu/Ile4-Asp5-Leu6-Val7 peptide and a C14 and C15 *β*-hydroxy fatty acid chain, respectively. **e** Surfactin precursor ions ion [M + H]^+^ at *m/z* 1008 at retention time 6.89 min containing a Glu1-Leu/Ile2-Leu3-Leu/Ile4-Asp5-Leu6-Leu/Ile7 peptide and a C12 *β*-hydroxy fatty acid chain

Figure 5b showed the LC–ESI–MS/MS spectrum of species observed as protonated ion at *m/z* 1022 at retention time 8.11 min. Then the sequence could be surmised as C_15_*β*-OH fatty acid-Glu^1^-Val ^2^-Leu^3^-Val^4^-Asp^5^-Leu^6^-Leu/Ile^7^. Thus, a protonated ion *m/z* at 1008 at retention time 8.46 min was assigned as homologs with C_14_*β*-OH fatty acid (Fig. 5a).

In the same way, we inferred the sequence of the precursor ion at *m/z* 1008 at retention time 8.40 min and 1022 at retention time 9.16 min were C_14_*β*-OH fatty acid-Glu^1^-Val^2^-Leu^3^-Leu/Ile^4^-Asp^5^-Leu^6^-Val^7^ and C_15_*β*-OH fatty acid-Glu^1^-Val^2^-Leu^3^-Leu/Ile ^4^-Asp^5^-Leu^6^-Val^7^, respectively (Fig. 5c, d).

A seventh surfactin isoform of LC–ESI–MS/MS spectrum of species observed as protonated ion was showed in Fig. 5e with a peptide sequence of Glu^1^-Leu/Ile^2^-Leu^3^-Leu/Ile^4^-Asp^5^-Leu^6^-Leu/Ile^7^ and a C_12_*β*-hydroxyl fatty acid chain with no Val.

**Fig. 6:**
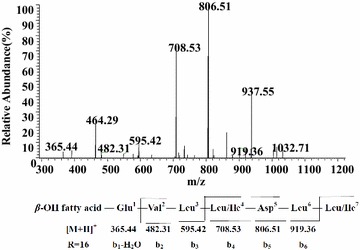
LC-ESI–MS/MS spectrum of esperin precursors ion [M + H]^+^ at *m/z* 1050 at retention time 4.68 min containing a Glu1-Leu/Ile2-Leu3-Leu/Ile4-Asp5-Leu6-Val7 peptide and a C16 *β*-hydroxy fatty acid chain

Figure 6 displayed the LC–ESI–MS/MS spectrum of the precursor ion at *m/z* 1050 at retention time 4.68 min. It could be concluded that it was a kind of esperin with the sequence could be surmised as Val^2^-Leu^3^- Leu/Ile^4^-Asp^5^-Leu^6^-Leu/Ile^7^ with a C_16_*β*-OH fatty acid based on its chromatographic behavior and fragmentation pattern. In the same way, we can conclude that the sequence of the precursor ion at *m/z* 1050 at retention time 5.12 min was C_16_*β*-OH fatty acid-Glu^1^-Leu/Ile^2^-Leu^3^-Leu/Ile^4^-Asp^5^-Leu^6^-Val^7^ (Additional file 1: Figure S4), an esperin, too.

**Fig. 7:**
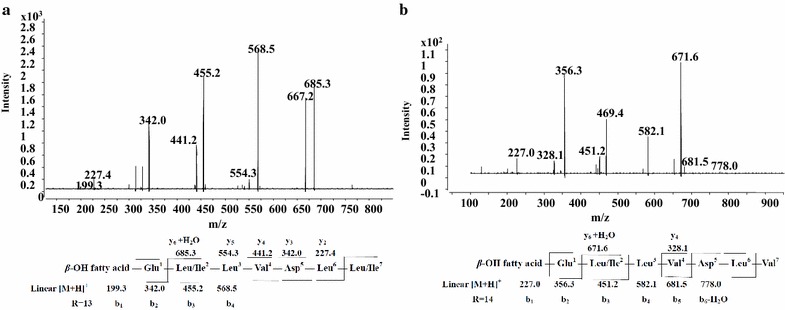
LC-ESI–MS/MS spectrum of the linear surfactin precursors. **a** Linear surfactin precursors ion [M + H]^+^ at *m/z* 1026 at retention time 5.26 min containing a Glu1-Leu/Ile2-Leu3-Val4-Asp5-Leu6-Leu/Ile7 peptide and a C13 *β*-hydroxy fatty acid chain. **b** Linear surfactin precursor ions [M + H]^+^ at *m/z* 1026 at retention time 5.53 min containing a Glu1-Leu/Ile2-Leu3-Val4-Asp5-Leu6-Val7 peptide and a C14 *β*-hydroxy fatty acid chain

As it is more, Fig. 7a showed the LC–ESI–MS/MS spectrum of species observed as protonated ion at *m/z* 1026 at retention time 5.26 min. We can conclude it was a linear surfactin with a peptide sequence of Glu^1^-Leu/Ile ^2^-Leu^3^-Val^4^-Asp^5^-Leu^6^-Leu/Ile^7^ and a C_13_*β*-hydroxyl fatty acid chain. In addition, the protonated ion *m/z* at 1012 at retention time 4.79 min (Additional file 1: Figure S5a), 1040 at retention time 5.89 min (Additional file 1: Figure S5b), 1054 at retention time 6.36 min (Additional file 1: Figure S5c) and 1068 at retention time 7.19 min (Additional file 1: Figure S5d) were assigned as homologs with C_11_, C_13_, C_14_, C_15_*β*-OH fatty acid. Using the same method, we can easily identify the protonated ion at *m/z* 1026 at the retention time 5.53 min was a linear surfactin possessing a peptide sequence of Glu^1^-Leu/Ile^2^-Leu^3^-Val^4^-Asp^5^-Leu^6^-Val^7^ and a C_14_*β*-hydroxyl fatty acid chain (Fig. 7b).

The protonated ion species of surfactin family containing with surfactins, esperins and linear surfactins isoforms characterized from the culture supernatants of marine *B. megatherium* are summarized in Table 1.

**Table 1:** Assignment of the structures of lipopeptides by LC–ESI–MS/MS in this study

Peak	Mass (M/S)	Rt (min)	Family	Assignment	Sequence
1	994	6.50	Surfactin	C_12_[M + H]^+^	
2	1008	6.98		C_13_[M + H]^+^	
3	1022	8.05		C_14_[M + H]^+^	
4	1036	9.12		C_15_[M + H]^+^	
5	1050	9.38		C_16_[M + H]^+^	
6	994	6.38		C_12_[M + H]^+^	
7	1008	6.94		C_13_[M + H]^+^	
8	1022	8.17		C_14_[M + H]^+^	
9	1036	8.63		C_15_[M + H]^+^	
10	1050	9.63		C_16_[M + H]^+^	
11	994	6.42		C_12_[M + H]^+^	
12	1008	6.89		C_13_[M + H]^+^	
13	1022	8.11		C_14_[M + H]^+^	
14	1036	8.69		C_15_[M + H]^+^	
16	1008	8.40		C_14_[M + H]^+^	
17	1022	9.35		C_15_[M + H]^+^	
18	1008	8.46		C_14_[M + H]^+^	
19	1022	9.22		C_15_[M + H]^+^	
20	1008	8.40	Surfactin	C_14_[M + H]^+^	
21	1022	9.16		C_15_[M + H]^+^	
22	1008	6.89		C_12_[M + H]^+^	
23	1050	4.68	Esperin	C_16_[M + H]^+^	
24	1050	5.12		C_16_[M + H]^+^	
25	1012	4.81	Surfactin	Linear C_12_[M + H]^+^	
26	1026	5.26		Linear C_13_[M + H]^+^	
27	1040	5.87		Linear C_14_[M + H]^+^	
28	1054	6.39		Linear C_15_[M + H]^+^	
29	1068	7.17		Linear C_16_[M + H]^+^	
30	1026	5.53	Surfactin	Linear C_14_[M + H]^+^	
31	1031	6.98	Bacillomycin D	C_14_[M + H]^+^	
32	1045	7.95		C_15_[M + H]^+^	
33	1451	10.94	Fengycin A	C_15_[M + H]^+^	
34	1465	10.82		C_16_[M + H]^+^	
35	1479	12.63		C_17_[M + H]^+^	
36	740	12.69		C_17_[M + 2H]^2+^	
37	1493	13.01		C_18_[M + H]^+^	
38	747	15.09		C_18_[M + 2H]^2+^	
39	1479	12.71	Fengycin B	C_15_[M + H]^+^	
40	740	12.61		C_15_[M + 2H]^2+^	
41	1493	12.92		C_16_[M + H]^+^	
42	747	13.15		C_16_[M + 2H]^2+^	
43	1467	8.66	Fengycin A	Linear C_15_[M + H]^+^	
44	1481	8.83		Linear C_16_[M + H]^+^	
45	1495	12.36		Linear C_17_[M + H]^+^	
46	1495	12.36	Fengycin B	Linear C_15_[M + H]^+^	
47	1509	13.87		Linear C_16_[M + H]^+^	

### LC–ESI–MS/MS characterisation of iturin lipopeptides

**Fig. 8:**
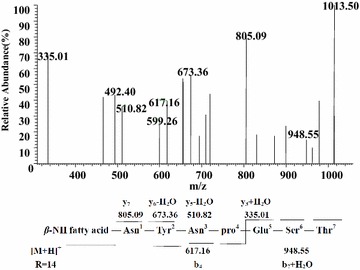
LC-ESI–MS/MS spectrum of the bacillomycin D precursors ion [M + H]^+^ at *m/z* 1031 at retention time 6.98 min containing a Asn1-Tyr2-Asn3-pro4-Glu5-Ser6-Thr7 peptide and a C14 *β*-amino fatty acid chain

Figure 8 displayed the LC–ESI–MS/MS spectrum of the precursor ion at *m/z* 1031 with appearance of fragment ions at retention time 6.98 min. A series y^+^ fragment at *m/z* 317 (+H_2_O, 335)(Thr^7^-Ser^6^-Glu^5^),528 (−H_2_O, 510)(Thr^7^-Ser^6^-Glu^5^-Pro^4^-Asn^3^), 691 (−H_2_O, 673)(Thr^7^-Ser^6^-Glu^5^-Pro^4^-Asn^3^-Tyr^2^-Asn^1^) and 805 (Thr^7^-Ser^6^-Glu^5^-Pro^4^-Asn^3^-Tyr^2^-Asn^1^-*β*-amino fatty acid) were found in the MS_2_ spectrum of *m*/*z* 1031. The b^+^ fragment ion at *m/z* 617 (*β*-amino fatty acid-Asn^1^-Tyr^2^-Asn^3^) and 948 (*β*-amino fatty acid-Asn^1^-Tyr^2^-Asn^3^-Pro^4^-Glu^5^-Ser^6^) were found, too. According to these typical CID fragments, the sequence could be deduced as C_14_*β*-amino fatty acid chain-Asn^1^-Tyr^2^-Asn^3^-Pro^4^-Glu^5^-Ser^6^-Thr^7^, which was assigned as bacillomycin D homologs.

The MS/MS spectrum of other protonated precursor ions at *m/z* 1045 at retention time 7.95 min (Additional file 1: Figure S6) also yielded similar profile of product ions was proved to be homologs possessing the same amino acid sequence but different C_15_*β*-amino fatty acids.

The protonated ion species of bacillomycin D homologs characterized from the culture supernatants of marine *B. megaterium* are summarized in Table 1.

### LC/ESI–MS/MS characterisation of fengycin lipopeptides

**Fig. 9:**
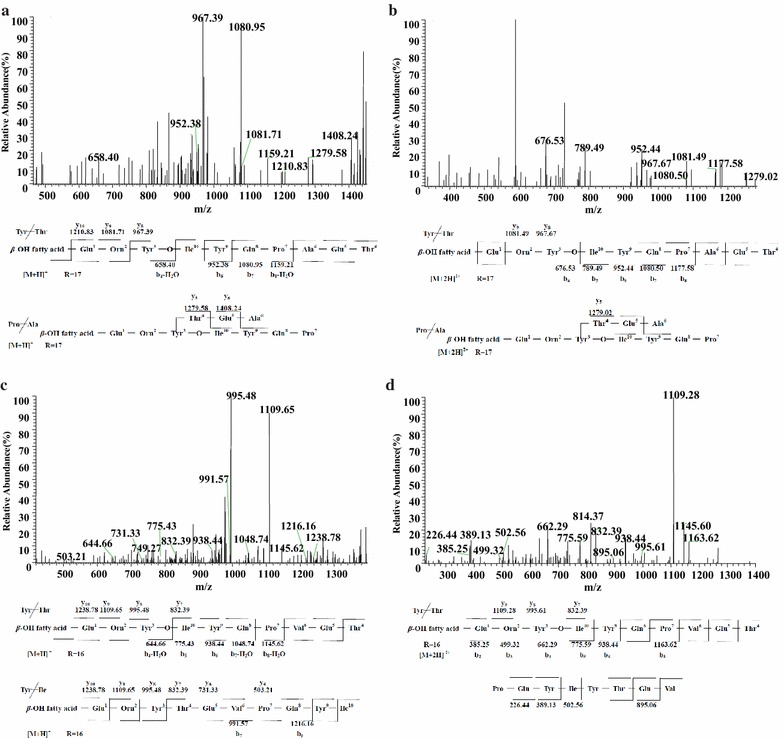
LC-ESI–MS/MS spectrum of the fengycin precursors. **a** Fengycin A precursors ion [M + H]^+^ at *m/z* 1479 at retention time 12.63 min containing a Glu1-Orn2-Tyr3-Thr4-Glu5-Ala6-Pro7-Gln8-Tyr9-Ile10 peptide and a C17 *β*-hydroxy fatty acid chain. **b** Fengycin A precursors ion [M + 2H]2^+^ at *m/z* 740 at retention time 12.69 min containing a C17 *β*-hydroxy fatty acid chain. **c** Fengycin B precursors ion [M + H]^+^ at *m/z* 1493 at retention time 12.92 min containing a Glu1-Orn2-Tyr3-Thr4-Glu5-Val6-Pro7-Gln8-Tyr9-Ile10 peptide and a C16 *β*-hydroxy fatty acid chain. **d** Fengycin B precursors ion [M + 2H]^2+^ at *m/z* 747 at retention time 13.15 min containing a C16 *β*-hydroxy fatty acid chain

Figure 9a displayed the LC–ESI–MS/MS spectrum of the precursor ion at *m/z* 1479 with appearance of fragment ions at retention time 12.63 min. The results showed the appearance of b^+^ product ions at *m/z* 1479 → 1177(−H_2_O, 1159) → 1080 → 952 → 676(−H_2_O, 658) were assigned as the losses of (Thr^4^-Glu^5^-Ala^6^)-Pro^7^-Gln^8^-(Tyr^9^-Ile^10^) and the y^+^ product ions at *m/z* 1210 → 1081 → 967 were corresponded to the neutral losses of side-chain Glu^1^–Orn^2^. These fragment ions were formed upon cleavage at Tyr^3^ and Thr^4^ in the middle of molecule. A third series fragment ions at *m/z* 1408 and 1279 meant the losses of Ala^6^ and Glu^5^- Ala^6^, respectively. This fragment series suggested there was another ring cleavage site between Ala^6^ and Pro^7^ of fengycin A homolog.

Figure 9b showed the LC–ESI–MS/MS spectrum of the precursor ion [M + 2H]^2+^ at *m/z* 740 with appearance of fragment ions at retention time 12.69 min. Dissociation yielded b^+^ ions at *m/z* 1479 → 1177 → 1080 → 952 → 789 → 676 were surmised as the losses of (Thr^4^-Glu^5^-Ala^6^)-Pro^7^-Gln^8^-Tyr^9^-Ile^10^ and y^+^ ions at *m/z* 1081 and 967 were corresponded to the neutral loses of *β*-hydroxyl fatty acid chain-Glu^1^ and *β*-hydroxyl fatty acid chain-Glu^1^-Orn^2^, respectively. From the product ions, [M + H]^+^ at *m/z* 1479 and [M + 2H]^2+^ at *m/z* 734 were with the same structure, it could be concluded that it was a kind of fengycin A with the sequence Glu^1^–Orn^2^-Tyr^3^-Thr^4^-Glu^5^-Ala^6^-Pro^7^-Gln^8^-Tyr^9^-Ile^10^ with a C_17_*β*-OH fatty acid based on its chromatographic behavior and fragmentation pattern.

In addition, the protonated ion *m/z* at 1451 at retention time 10.94 min (Additional file 1: Figure S7a), 1465 at retention time 10.82 min (Additional file 1: Figure S7b), 1493 at retention time 13.01 min (Additional file 1: Figure S7c) and [M + 2H]^2+^ at *m/z* 747 at retention time 15.09 min (Additional file 1: Figure S7d) with multiples of 14 Da difference in their molecular ion species were proved to be Fengycin A homologs with the same amino acid sequence but different C_15_, C_16_, C_18_ and C_18_*β*-OH fatty acids chain.

A second fengycin isoforms of LC–ESI–MS/MS spectrum of species observed as protonated ion were showed on Fig. 9c, d and Additional file 1: Figure S8. Then the sequence of Fig. 9c could be concluded based on the fragmentation profile as C_16_*β*-OH fatty acid-Glu^1^–Orn^2^-Tyr^3^-Thr^4^-Glu^5^-Val^6^-Pro^7^-Gln^8^-Tyr^9^-Ile^10^. Figure 9d showed fragment ions of precursor ion [M + 2H]^2+^ at *m/z* 747 at retention time 13.15 min. It turned out that it has the same structure with *m/z* 1493 at retention time 12.92 min. In addition to this, a protonated ion *m/z* at 1479 at retention time 12.71 min (Additional file 1: Figure S8a) and [M + 2H]^2+^ at *m/z* 740 at retention time 12.61 min (Additional file 1: Figure S8b) were assigned as fengycin B homologs with C_15_*β*-OH fatty acid.

**Fig. 10:**
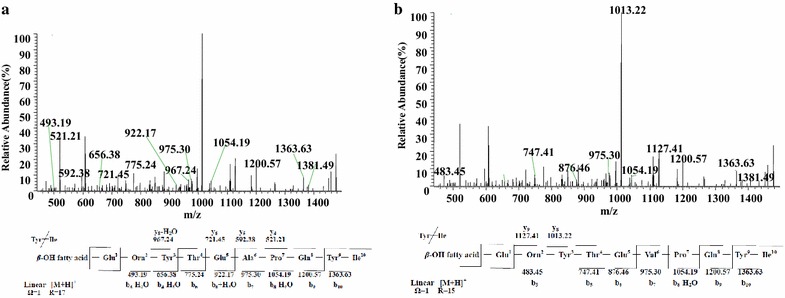
LC-ESI–MS/MS spectrum of the linear fengycin precursors. **a** Linear fengycin A precursors ion [M + H]^+^ at *m/z* 1495 containing a Glu1-Orn2-Tyr3-Thr4-Glu5-Ala6-Pro7-Gln8-Tyr9-Ile10 peptide and a C17 *β*-hydroxy fatty acid chain with a double bond. **b** Linear fengycin B precursors ion [M + H]^+^ at *m/z* 1495 containing a Glu1-Orn2-Tyr3-Thr4-Glu5-Val6-Pro7-Gln8-Tyr9-Ile10 peptide and a C15 *β*-hydroxy fatty acid chain with a double bond

Figure 10a showed the LC–ESI–MS/MS spectrum of species observed as protonated ion at *m/z* 1495 at retention time 12.36 min. We concluded it is a linear fengycin A with a peptide sequence of Glu^1^-Orn^2^-Tyr^3^-Thr^4^-Glu^5^-Ala^6^-Pro^7^-Gln^8^-Tyr^9^-Ile^10^ and a C_17_*β*-hydroxyl fatty acid chain with a double bond based on its chromatographic behavior and fragmentation pattern. The protonated ion *m/z* at 1467 at retention time 8.66 min (Additional file 1: Figure S9a) and 1481 at retention time 8.79 min (Additional file 1: Figure S9b) with characteristic fragment ions *m/z* 967 and 1081 were assigned as linear fengycin A homologs with C_15_ and C_16_*β*-OH fatty acid with a double bond, respectively.

The LC–ESI–MS/MS spectrum of species observed as protonated ion at *m/z* 1495 at retention time 12.36 min had a second series of fragmentation ions were showed that it contained a peptide sequence of Glu^1^-Orn^2^-Tyr^3^-Thr^4^-Glu^5^-Val^6^-Pro^7^-Gln^8^-Tyr^9^-Ile^10^ and a C_15_*β*-hydroxyl fatty acid chain with a double bond (Fig. 10b). Besides, a protonated ion *m/z* at 1509 at retention time 13.87 min with characteristic fragment ions *m*/*z* 995 was assigned as linear fengycin B homologs with C_16_*β*-OH fatty acid with a double bond (Additional file 1: Figure S10).

The protonated ion species of fengycin family containing with fengycin A, B and linear fengycin isoforms characterized from the culture supernatants of marine *B. megaterium* are summarized in Table 1.

## Discussion

LC–ESI–MS was performed in order to investigate the nature of the compounds in the methanol extract after a two-step ultrafiltration. Different families of lipopeptides with surfactins, fengycins and iturins can be eluted by 85–100, 50–70, 40–50 % acetonitrile in water, respectively, in reversed phase high performance liquid chromatography (RP-HPLC) system (Yang et al. 2015). The three families of molecules were successively separated and characterised by LC–ESI–MS in positive full scan mode.

Surfactin family contains more than 20 different lipopeptides such as lichenysin, pumilacidin, esperin and surfactin. Esperin is coincided with those established surfactin for the composition and the amino acid residues, except that there is a smaller peptide ring formed by the fifth l-Asp in the esperin, and not by the seventh Leu-residue like surfactin (Kalinovskaya et al. 1995; Thomas and Ito 1969). Surfactins have the common following structural traits: a heptapeptide with a chiral sequence LLDLLDL interlinked with a *β*-hydroxy fatty acid and with a d-Leu in position 3 and 6 and a l-Asp in position 5. Amino acid residues in position 2, 4 and 7 belong to the amino acids group including Val, Leu and Ile (Jacques 2011; Hue et al. 2001). With a Gln in the position 1 are named lichenysin which are different from surfactin with the Glu in the position 1 (Qiu et al. 2014). Some of the isoforms had the same *m*/*z* by ESI–MS, but they were in different HPLC fraction. Thus, it is necessary to precisely identify their structure by MS/MS, especially for the amino acid sequence of the peptide portion of the molecules (Yang et al. 2015).

According to the results of LC–MS, ten major fractions with different molecular weight were used as precursor ions for further ESI–MS/MS analysis. In details, the precursor ions at *m/z* 994, 1008, 1022, 1036 and 1050 were hypothesized to be a series of homologue molecules of surfactins with 14 or multiples of 14 Da difference in their molecular ion species.

From the results of LC–ESI–MS/MS of Figs. 4 and 5, the presence of fragment ions at *m/z* 685, 671 or 681 are the common peaks in CID spectrum of surfactin isoforms ion. The amino acids sequences at *m/z* 671, 685 and 681 are [(H)Leu/Ile^2^-Leu^3^-Val^4^-Asp^5^-Leu^6^-Val^7^(OH) + H]^+^/[(H)Val ^2^-Leu^3^-Val^4^-Asp^5^-Leu^6^-Leu/Ile^7^(OH) + H]^+^/[(H)Val^2^-Leu^3^-Leu/Ile^4^-Asp^5^-Leu^6^-Val^7^(OH) + H]^+^, [(H)Leu/Ile^2^-Leu^3^-Val^4^-Asp^5^-Leu^6^- Leu/Ile^7^(OH) + H]^+^/[(H)Val^2^-Leu^3^-Leu/Ile^4^-Asp^5^-Leu^6^-Leu/Ile ^7^(OH) + H]^+^/[(H)Leu/Ile^2^-Leu^3^-Leu/Ile^4^-Asp^5^-Leu^6^-Val^7^(OH) + H]^+^ and [(H)Leu/Ile^2^-Leu^3^-Leu/Ile^4^-Asp^5^-Leu^6^-Leu/Ile^7^(OH) + H]^+^, respectively. Thus, the fragment ions at *m/z* 685, 671 or 681 are characteristic marker ions for identification of surfactins.

Through LC–ESI–MS, the chromatogram for surfactin family showed two unique peaks at retention time 4.68 and 5.12 min corresponding to molecules [M + H]^+^*m/z* 1050. Their retention times were much less than the surfactin standard time, thus, according to the principle of similar miscibility, it showed that these molecules with stronger polarity. Figure 6 showed the dissociation yielded b^+^ ions at *m/z* 1050 (−H_2_O, 1032) → 919 → 806 → 708 → 595 → 482 → 383 (−H_2_O, 365) were surmised as the losses of (OH)Leu/Ile^7^-Leu^6^-Asp(-OH)^5^-Leu/Ile^4^-Leu^3^-Val^2^ from the C terminus. Therefore, we can infer its amino acids sequence is Glu^1^-Val^2^-Leu^3^-Leu/Ile^4^-Asp^5^-Leu^6^-Leu/Ile^7^, which is the same with surfactin. But, its fragment ions didn’t have the characteristic fragment ion *m/z* 685 (Figs. 4, 5) which can be inferred it wasn’t surfactin. Simultaneously, the peak from *m/z* 806 to 708 in Fig. 6 was formed by loss of the Asp(−OH)^5^ from molecule, which confirmed that the carboxyl group of Asp and the hydroxyl group of the aliphatic part formed the ester structure in the esperin molecule, and this is another evidence of our inferences. The most noteworthy is that the fragment ions of it at *m/z* 450 were matched with the losses of H^+^(OH)Val^7^-Leu^6^-Asp^5^-C_16_*β*-OH fatty acid from the C terminus which further improved the carboxyl group of Asp and the hydroxyl group of the aliphatic part formed the ester structure in the esperin molecule.

According to LC–ESI–MS, before the elution of surfactins (4.5–7.2 min), several peaks appeared that correspond to unknown compounds at *m/z* 1012 at retention time 4.79 min, 1026 at retention time 5.26 min, 1026 at retention time 5.53 min, 1040 at retention time 5.89 min, 1054 at retention time 6.36 min and 1068 at retention time 7.19 min. What a coincidence is that, compared with the surfactins at *m/z* 994, 1008, 1022, 1036 and 1050, respectively, the *m/z* precursor molecules are 18 Da bigger than surfactins correspondingly. As we know, the relative molecular mass of the H_2_O was just 18. We can conclude they were linear surfactins based on their chromatographic behavior and fragmentation pattern.

Though iturin has the limited antiviral and antibacterial activity, but it displays strong antifungal activity (Maget-Dana and Peypoux 1994). Bacillomycin D, closely related variants with iturin, is famous for the high activity against *Aspergillus flavus* (Moyne et al. 2001). According to the results of LC–MS, the most intense fractions with different molecules at *m/z* 1031 and 1045 were used as precursor ions by ESI–MS/MS analyzes for further peptide sequence determination and some others [M + H]^+^ at *m/z* 1059 and 1073. They were hypothesized to be a series of homologous molecules of bacillomycin D with multiples of 14 Da difference in their molecular ion species.

Fengycin homologs are divided into two different types (fengycin A and B) by their amino acid sequence. The fengycin A has the Ala at position 6, while Val for fengycin B (Vater et al. 2002). Fengycins (plipastatin if Tyr^9^ is D-configured) (Raaijmakers et al. 2010), the configuration of amino acid sequence is LDLDLDDLLDL with a lactone bond connecting l-Tyr to l_-_Ile, includes fengycins A, B and relevant homologs with the difference in the amino acid at position 6. They are macrolactone rings with the Tyr side chain at position 3 of the peptide sequence and the C-terminal residue yielding an internal ring by an ester bond.

From the above, the results (Fig. 9a, b; Additional file 1: Figure S7) showed the appearance of product ions at m/z 1081 and 967 from a series of precursor ions and the results (Fig. 9c, d and Additional file 1: Figure S8) showed the appearance of product ions at *m/z* 1109 and 995 from a series of precursor ions. The molecular weights of two group peaks (1081 and 1109, 967 and 995) were differed by 28 Da, suggesting a difference in the amino acid composition of the peptide (Ala or Val). Thus, *m/z* 1080 and 967 are characteristic ions for fengycin A and *m/z* 1109 and 995 are characteristic ions for fengycin B with the information of specific octapeptide ring ions.

The result (Fig. 10a) showed the b^+^ fragment ions at *m/z* 1495(−H_2_O, 1477) → 1363 → 1200 → 1072(-H_2_O, 1054) → 975 → 904(+H_2_O, 922) → 775 → 674(−H_2_O, 656) → 511(−H_2_O, 493) matched with the losses of Tyr^3^-Thr^4^-Glu^5^-Ala^6^-Pro^7^-Gln^8^-Tyr^9^-Ile^10^. Its y^+^ fragment ions at *m/z* 985(−H_2_O, 967) → 721 → 592 → 521 meant the losses of (Tyr^3^-Thr^4^)-Glu^5^-Ala^6^ in the middle of precursor ion and the peak of 967 is the characteristic fragment ion of fengycin A. It was 16 Da bigger than *m*/*z* 1479. We concluded it was a linear fengycin A with a peptide sequence of Glu^1^-Orn^2^-Tyr^3^-Thr^4^-Glu^5^-Ala^6^-Pro^7^-Gln^8^-Tyr^9^-Ile^10^ and a C_17_*β*-hydroxyl fatty acid chain with a double bond. The result (Fig. 10b) showed the b^+^ fragment ions at *m/z* 1495(−H_2_O, 1477) → 1363 → 1200 → 1072(−H_2_O, 1054) → 975 → 876 → 747 → 483 revealed the losses of Tyr^3^-Thr^4^-Glu^5^-Val^6^-Pro^7^-Gln^8^-Tyr^9^-Ile^10^ from the C terminus. Its y^+^ fragment ions at *m/z* 1127 → 1013 meaned the losses of Orn^2^, suggesting the precursor ion (a linear fengycin B) possessed a peptide sequence of Glu^1^-Orn^2^-Tyr^3^-Thr^4^-Glu^5^-Val^6^-Pro^7^-Gln^8^-Tyr^9^-Ile^10^ and a C_15_*β*-hydroxyl fatty acid chain with a double bond.

LC–ESI–MS/MS was performed after a two-step ultrafiltration in order to investigate the nature of the compounds in the methanol extract of marine *B. megaterium*. The three families of molecules were successively separated and characterised by LC–ESI–MS in positive full scan mode. LC–ESI–MS/MS analysis precisely elucidated the different amino sequence of isoforms of iturin, surfactin and fengycin families. Collectively, we have established seven kinds of cyclic isoforms of surfactin with a series of homologs (Table 1). The typical difference among these surfactins is amino acid residues in position 2, 4 and 7 belong to the amino acids group including Val, Leu and Ile. Ile or Leu is difficult to distinguish with mass spectrometry. It is worth noting that a variety of linear isoforms of surfactin were identified with relatively high abundance. In addition to this, two kinds of cyclic isoforms of esperin were separated and identified, which was consistent with others (Kalinovskaya et al. 1995). The peptide sequence of bacillomycin D homologs which exhibited potent inhibitory effect on *A. flavus* were also identified. In this study, in addition to the popular cyclic fengycin A and B with the difference in the amino acid at position 6 of the peptide moiety, two kinds of linear fengycin A and B with a double bond were characterized for the first time.

In summary, this is the first report of an *Bacillus* strain co-producing so many variants of lipopeptides including cyclic surfactin, linear surfactin, esperin, bacillomycin D, cyclic fengycin A, linear fengycin A, cyclic fengycin B and linear fengycin B. The structure of different lipopeptides has a good reference for the future research.

## Additional file

10.1186/s13568-016-0252-6 Additional figures.

## Abbreviations

LC–ESI–MS/MS: liquid chromatography-electronic spray ionization-tandem mass spectrometry
CID: collision-induced dissociation
CMC: critical micelle concentration
CGMCC: China General Microbiological Culture Collection Center
tR: retention time
RP-HPLC: reversed phase high performance liquid chromatography
