# Supplementary material for: Identification of lipopeptides in Bacillus megaterium by two-step ultrafiltration and LC–ESI–MS/MS
Source: AMB Express. 2016 Sep 17;6:79. doi: 10.1186/s13568-016-0252-6 (PMC5026979; doi:10.1186/s13568-016-0252-6)
Supplement: Supplementary file 1 — 10.1186/s13568-016-0252-6 Additional figures. [file 13568_2016_252_MOESM1_ESM.doc]

**Identification of lipopeptides in *Bacillus megaterium* by two-step ultrafiltration and LC-ESI-MS/MS**

Yunxiao Ma^1^, Qing Kong^1,*^, Chong Qin^2^, Yulin Chen^1^, Yujie Chen^1^, Ruihuan Lv^1^, Guanghui Zhou^1^

^1^School of Food Science and Engineering, Ocean University of China, Qingdao, Shandong, China

^2^Department of Medicinal Chemistry, College of Pharmacy, Univesity of Michigan, Ann Arbor, Michigan, USA

**Running title:** Identification of lipopeptides in *B. megaterium*

***Correspondence:** Dr. Qing Kong, School of Food Science and Engineering, Ocean University of China, Qingdao. Yushan Road 5, 62 Building, Qingdao, Shandong 266003, China

**E-mail:** kongqing@ouc.edu.cn
**Tel:** +86-532-8203-1851

**Fax:** +86-532-8203-2272

**a b**

**300**

**400**

**500**

**600**

**700**

**800**

**900**

**1000**

**m/z**

**0**

**10**

**20**

**30**

**40**

**50**

**60**

**70**

**80**

**90**

**100**

**685.37**

**667.21**

**568.11**

**895.22**

**990.63**

**341.99**

**323.87**

**454.73**

**782.32**

**554.31**

**Relative Abundance (%)**


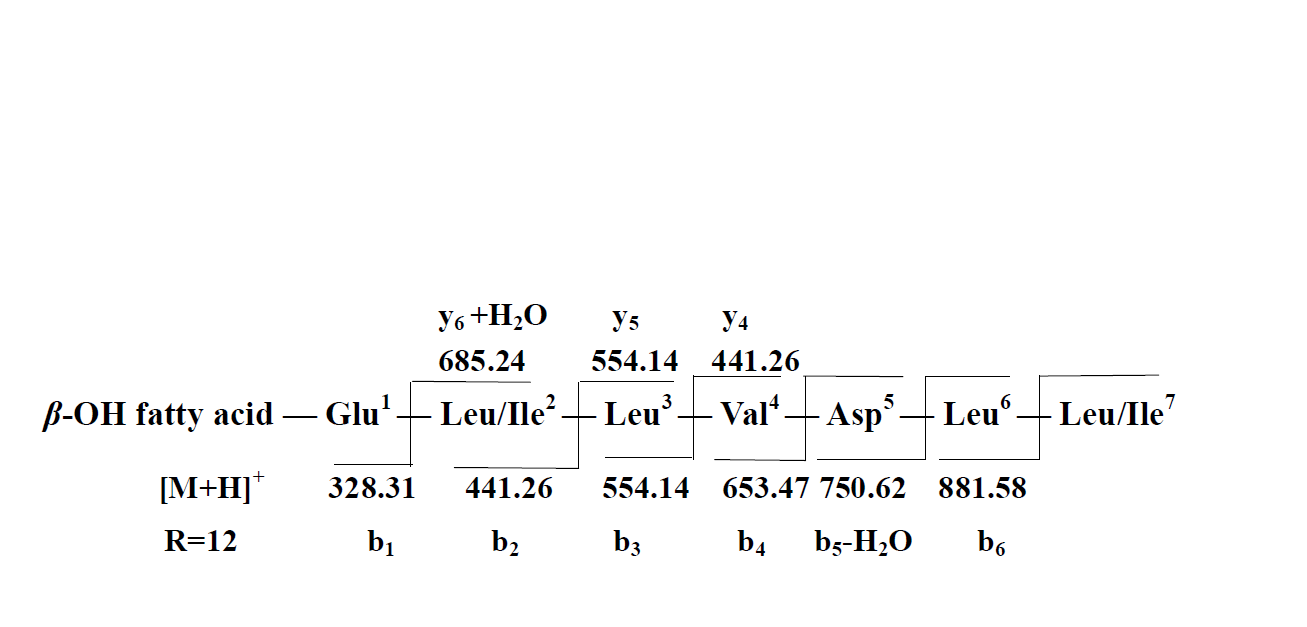


**Relative Abundance (%)**

**300**

**400**

**500**

**600**

**700**

**800**

**900**

**1000**

**m/z**

**0**

**10**

**20**

**30**

**40**

**50**

**60**

**70**

**80**

**90**

**100**

**685.24**

**863.41**

**441.26**

**554.14**

**881.58**

**976.12**

**310.12**

**653.47**

**536.10**

**750.62**

**328.31**

**c d**

**Relative Abundance (%)**

**400**

**500**

**600**

**700**

**800**

**900**

**1000**

**1100**

**1200**

**m/z**

**0**

**10**

**20**

**30**

**40**

**50**

**60**

**70**

**80**

**90**

**100**

**685.27**

**937.03**

**610.10**

**554.09**

**709.04**

**497.04**


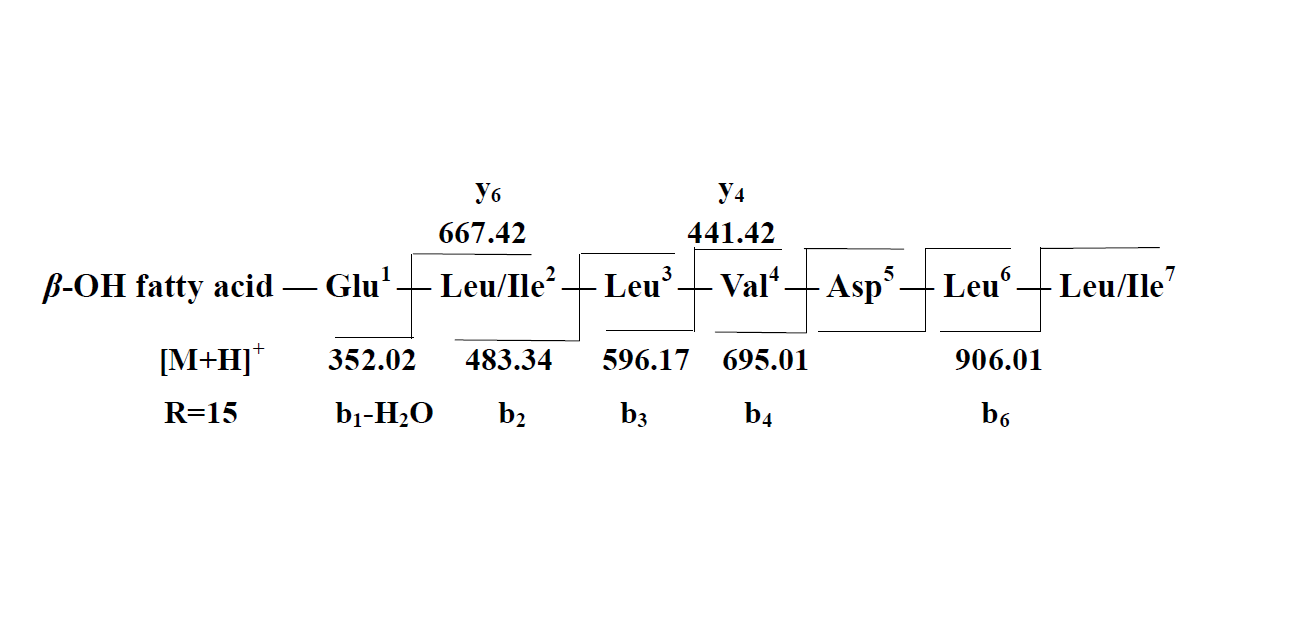


**Relative Abundance (%)**

**400**

**500**

**600**

**700**

**800**

**900**

**1000**

**0**

**10**

**20**

**30**

**40**

**50**

**60**

**70**

**80**

**90**

**100**

**685.16**

**596.17**

**667.42**

**578.25**

**906.01**

**483.34**

**991.48**

**441.42**

**695.01**

**352.02**

**m/z**

**Relative Abundance(%)**

Supplementary Fig. S1 LC-ESI-MS/MS spectrum of the surfactin precursors. a Surfactin precusors ion [M+H]^+^ at *m/z* 994 at retention time 6.50 min, containing a Glu^1^-Leu/Ile^2^-Leu^3^-Val^4^-Asp^5^-Leu^6^-Leu/Ile^7^ peptide and a C_12_ *β*-hydroxy fatty acid chain. b Surfactin precursors ion [M+H]^+^ at *m/z* 1,008 at retention time 6.98 min containing a C_13_ *β*-hydroxy fatty acid chain. c Surfactin precursors ion [M+H]^+^ at *m/z* 1,036 at retention time 9.12 min containing a C_15_ *β*-hydroxy fatty acid chain. d Surfactin precursors ion [M+H]^+^ at *m/z* 1,050 at retention time 9.38 min containing a C_16_ *β*-hydroxy fatty acid chain.

**a b**


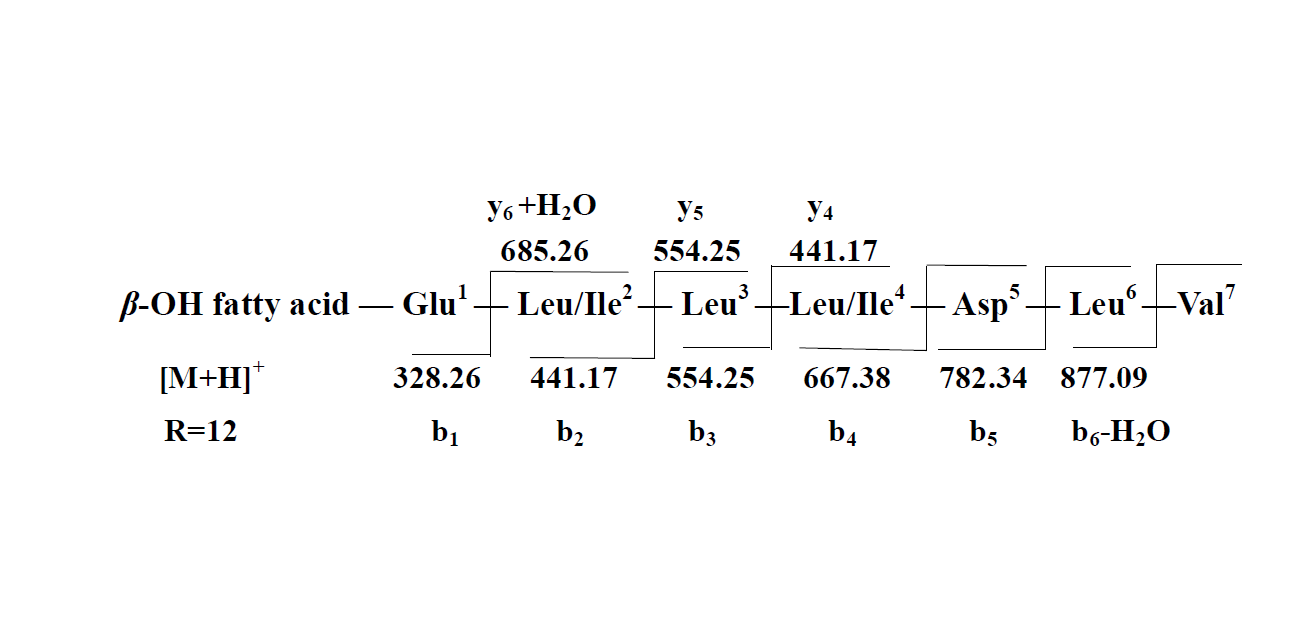


**Relative Abundance (%)**

**350**

**400**

**450**

**500**

**550**

**600**

**650**

**700**

**750**

**800**

**850**

**900**

**950**

**m/z**

**0**

**10**

**20**

**30**

**40**

**50**

**60**

**70**

**80**

**90**

**100**

**976.38**

**667.38**

**554.25**

**572.34**

**877.09**

**328.26**

**685.26**

**441.17**

**782.34**

**Relative Abundance (%)**

**300**

**350**

**400**

**450**

**500**

**550**

**600**

**650**

**700**

**750**

**800**

**850**

**900**

**950**

**m/z**

**0**

**10**

**20**

**30**

**40**

**50**

**60**

**70**

**80**

**90**

**100**

**685.26**

**667.35**

**990.55**

**568.23**

**455.16**

**892.46**

**441.17**

**910.47**

**342.06**

**681.45**

**554.25**

**c d**

**Relative Abundance (%)**

**400**

**500**

**600**

**700**

**800**

**900**

**1000**

**m/z**

**0**

**10**

**20**

**30**

**40**

**50**

**60**

**70**

**80**

**90**

**100**

**685.36**

**596.15**

**667.42**

**1018.46**

**483.26**

**554.17**

**370.35**

**441.26**

**352.27**

**709.27**

**806.52**

**937.57**

**Relative Abundance (%)**

**300**

**400**

**500**

**600**

**700**

**800**

**900**

**1000**

**1100**

**m/z**

**0**

**10**

**20**

**30**

**40**

**50**

**60**

**70**

**80**

**90**

**100**

**685.28**

**592.14**

**610.24**

**366.52**

**723.49**

**951.74**

**479.32**

**1032.64**

**423.18**

Supplementary Fig. S2 LC-ESI-MS/MS spectrum of the surfactin precursors. a Surfactin precusors ion [M+H]^+^ at *m/z* 994 containing a Glu^1^-Leu/Ile^2^-Leu^3^-Leu/Ile^4^-Asp^5^-Leu^6^-Val^7^ peptide and a C_12_ *β*-hydroxy fatty acid chain. b Surfactin precursors ion [M+H]^+^ at *m/z* 1,008 containing a C_13_ *β*-hydroxy fatty acid chain. c Surfactin precursors ion [M+H]^+^ at *m/z* 1,036 containing a C_15_*β*-hydroxy fatty acid chain. d Surfactin precursors ion [M+H]^+^ at *m/z* 1,050 containing a C_16_*β*-hydroxy fatty acid chain.

**a b**

**Relative Abundance (%)**

**300**

**350**

**400**

**450**

**500**

**550**

**600**

**650**

**700**

**750**

**800**

**850**

**900**

**950**

**m/z**

**0**

**10**

**20**

**30**

**40**

**50**

**70**

**80**

**90**

**100**

**990.51**

**685.47**

**568.19**

**895.34**

**536.36**

**423.38**

**441.24**

**667.24**

**782.61**

**455.20**

**342.13**

**554.15**

**60**

**Relative Abundance (%)**

**300**

**400**

**500**

**600**

**700**

**800**

**900**

**1000**

**m/z**

**0**

**10**

**20**

**30**

**40**

**50**

**60**

**70**

**80**

**90**

**100**

**685.32**

**863.23**

**976.44**

**635.34**

**667.32**

**881.58**

**328.05**

**568.14**

**427.51**

(b)

(a)

**c**

(c)

**Relative Abundance (%)**

**400**

**500**

**600**

**700**

**800**

**900**

**1000**

**m/z**

**0**

**10**

**20**

**30**

**40**

**50**

**60**

**70**

**80**

**90**

**100**

**685.30**

**810.24**

**1018.35**

**564.42**

**905.32**

**667.28**

**923.27**

**792.44**

**370.06**

**695.17**

**568.56**

Supplementary Fig. S3 LC-ESI-MS/MS spectrum of the surfactin precursors. a Surfactin precusors ion [M+H]^+^ at *m/z* 994 containing a Glu^1^-Val^2^-Leu^3^-Leu/Ile^4^-Asp^5^-Leu^6^-Leu/Ile^7^ peptide and a C_12_ *β*-hydroxy fatty acid chain. b Surfactin precursors ion [M+H]^+^ at *m/z* 1,008 containing a C_13_ *β*-hydroxy fatty acid chain. c Surfactin precursors ion [M+H]^+^ at *m/z* 1,036 containing a C_15_ *β*-hydroxy fatty acid chain.

**400**

**500**

**600**

**700**

**800**

**900**

**1000**

**m/z**

**0**

**10**

**20**

**30**

**40**

**50**

**60**

**70**

**80**

**90**

**100**

**820.54**

**806.45**

**1032.34**

**951.37**

**596.22**

**723.34**

**450.11**

**937.49**

**711.34**

**Relative Abundance (%)**

Supplementary Fig. S4 LC-ESI-MS/MS spectrum of the esperin precusors ion [M+H]^+^ at *m/z* 1,050 at retention time 5.12 min containing a Glu^1^-Val^2^-Leu^3^-Leu/Ile^4^-Asp^5^-Leu^6^-Leu/Ile^7^ peptide and a C_16_ *β*-hydroxy fatty acid chain.

**a b**

**0**

**0.2**

**0.4**

**0.6**

**0.8**

**1**

**1.2**

**1.4**

**1.6**

**1.8**

**441.30**

**227.1**

**685.5**

**199.3**

**328.0**

**554.3**

**m/z**

**200**

**250**

**300**

**350**

**400**

**450**

**500**

**550**

**600**

**650**

**700**

**342.2**

**x10^1^**

**Intensity**

**Intensity**

**x10^2^**

**-0.1**

**0**

**0.1**

**0.2**

**0.3**

**0.4**

**0.5**

**0.6**

**0.7**

**0.8**

**0.9**

**1**

**1.1**

**685.4**

**582.4**

**469.3**

**356.3**

**227.2**

**909.4**

**m/z**

**100**

**200**

**300**

**400**

**500**

**600**

**700**

**800**

**900**

**681.5**

**441.3**

**554.4**

**796.4**

**c d**

**Intensity**

**x10^2^**

**-0.1**

**0**

**0.1**

**0.2**

**0.3**

**0.4**

**0.5**

**0.6**

**0.7**

**0.8**

**0.9**

**1**

**1.1**

**384.2**

**227.1**

**497.3**

**441.2**

**610.4**

**685.4**

**m/z**

**50**

**100**

**150**

**200**

**250**

**300**

**350**

**400**

**450**

**500**

**550**

**600**

**650**

**700**

**750**

**800**

**255.3**

**342.2**

**Intensity**

**x10^2^**

**-0.1**

**0**

**0.1**

**0.2**

**0.3**

**0.4**

**0.5**

**0.6**

**0.7**

**0.8**

**0.9**

**1**

**1.1**

**685.5**

**596.5**

**483.4**

**441.3**

**370.3**

**227.2**

**667.4**

**342.1**

**m/z**

**200**

**300**

**400**

**500**

**600**

**700**

**241.3**

**554.4**

**695.6**

Supplementary Fig. S5 LC-ESI-MS/MS spectrum of the linear surfactin precursors. a Linear surfactin precusors ion [M+H]^+^ at *m/z* 1,012 containing a Glu^1^-Leu/Ile^2^-Leu^3^-Val^4^-Asp^5^-Leu6-Leu/Ile^7^ peptide and a C_12_ *β*-hydroxy fatty acid chain. b Linear surfactin precursors ion [M+H]^+^ at *m/z* 1,040 containing a C_14_ *β*-hydroxy fatty acid chain. c Linear surfactin precursors ion [M+H]^+^ at *m/z* 1,054 containing a C_15_ *β*-hydroxy fatty acid chain. d Linear surfactin precursors ion [M+H]^+^ at *m/z* 1,068 containing a C_16_ *β*-hydroxy fatty acid chain.

**Relative Abundance(%)**

**300**

**400**

**500**

**600**

**700**

**800**

**900**

**1000**

**m/z**

**0**

**10**

**20**

**30**

**40**

**50**

**60**

**70**

**80**

**90**

**100**

**688.15**

**931.44**

**1027.10**

**392.57**

**323.26**

**768.40**

Supplementary Fig. S6 LC-ESI-MS/MS spectrum of the bacillomycin D precursors ion [M+H]^+^ at *m/z* 1,045 at retention time 7.95 min containing a C_15_ *β*-amino fatty acid chain

.

**a**   **b**

**500**

**600**

**700**

**800**

**900**

**1000**

**1100**

**1200**

**1300**

**1400**

**m/z**

**0**

**10**

**20**

**30**

**40**

**50**

**60**

**70**

**80**

**90**

**100**

**1210.85**

**1052.52**

**804.53**

**1397.15**

**1250.07**

**1000**

**1100**

**1200**

**1300**

**1400**

**1500**

**1600**

**1700**

**m/z**

**0**

**10**

**20**

**30**

**40**

**50**

**60**

**70**

**80**

**90**

**100**

**967.60**

**1081.44**

**500**

**600**

**700**

**800**

**900**

**1000**

**1100**

**1200**

**1300**

**1400**

**m/z**

**0**

**10**

**20**

**30**

**40**

**50**

**60**

**70**

**80**

**90**

**100**

**1210.85**

**1052.52**

**804.53**

**1397.15**

**1250.07**

**1000**

**1100**

**1200**

**1300**

**1400**

**1500**

**1600**

**1700**

**m/z**

**0**

**10**

**20**

**30**

**40**

**50**

**60**

**70**

**80**

**90**

**100**

**967.60**

**1081.44**

**Relative Abundance (%)**

**Relative Abundance (%)**

**c d**

**500**

**600**

**700**

**800**

**900**

**1000**

**1100**

**1200**

**1300**

**1400**

**m/z**

**0**

**10**

**20**

**30**

**40**

**50**

**60**

**70**

**80**

**90**

**100**

**967.45**

**1094.26**

**527.13**

**1081.71**

**804.50**

**966.39**

**703.27**

**503.11**

**300**

**400**

**500**

**600**

**700**

**800**

**900**

**1000**

**1100**

**1200**

**1300**

**m/z**

**0**

**10**

**20**

**30**

**40**

**50**

**60**

**70**

**80**

**90**

**100**

**690.14**

**804.36**

**966.58**

**1081.54**

**1094.60**

**1173.47**

**967.21**

**527.03**

**Relative Abundance (%)**

**Relative Abundance (%)**

Supplementary Fig. S7 LC-ESI-MS/MS spectrum of the fengycin A precussors. a Fengycin A precusors ion [M+H]^+^ at *m/z* 1,451 containing a C_15_ *β*-hydroxy fatty acid chain. b Fengycin A precusors ion [M+H]^+^ at *m/z* 1,465 containing a C_16_ *β*-hydroxy fatty acid chain. c Fengycin A precusors ion [M+H]^+^ at *m/z* 1,493 containing a C_18_ *β*-hydroxy fatty acid chain. d Fengycin A precusors ion [M+2H]^2+^ at *m/z* 747 containing a C_18_ *β*-hydroxy fatty acid chain.

**Relative Abundance(%)**

**a b**

**Relative Abundance (%)**

**Relative Abundance (%)**

**300**

**400**

**500**

**600**

**700**

**800**

**900**

**1000**

**1100**

**1200**

**1300**

**1400**

**m/z**

**0**

**10**

**20**

**30**

**40**

**50**

**60**

**70**

**80**

**90**

**100**

**1091.56**

**1034.59**

**1149.38**

**977.01**

**666.31**

**1251.50**

**761.36**

**500**

**600**

**700**

**800**

**900**

**1000**

**1100**

**1200**

**1300**

**1400**

**m/z**

**0**

**10**

**20**

**30**

**40**

**50**

**60**

**70**

**80**

**90**

**100**

**1251.46**

**1149.45**

**995.37**

**761.66**

**1398.30**

**648.42**

**1109.55**

Supplementary Fig.S8 LC-ESI-MS/MS spectrum of the fengycin B precussors. a Fengycin B precusors ion [M+H]^+^ at *m/z* 1,479 containing a Glu^1^-Orn^2^-Tyr^3^-Thr^4^-Glu^5^-Val^6^-Pro^7^-Gln^8^-Tyr^9^-Ile^10^ peptide and a C_15_ *β*-hydroxy fatty acid chain. b Fengycin B precusors ion [M+2H]^2+^ at *m/z* 740 containing a C_15_ *β*-hydroxy fatty acid chain.

**a b**

**Relative Abundance (%)**

**Relative Abundance (%)**

**500**

**600**

**700**

**800**

**900**

**1000**

**1100**

**1200**

**1300**

**1400**

**m/z**

**0**

**10**

**20**

**30**

**40**

**50**

**60**

**70**

**80**

**90**

**100**

**967.33**

**1081.67**

**600**

**800**

**1000**

**1200**

**1400**

**1600**

**1800**

**2000**

**m/z**

**0**

**10**

**20**

**30**

**40**

**50**

**60**

**70**

**80**

**90**

**100**

**1081.62**

**949.66**

Supplementary Fig. S9 LC-ESI-MS/MS spectrum of the linear fengycin A precussors. a Linear fengycin A precusors ion [M+H]^+^ at *m/z* 1,467 containing a Glu^1^-Orn^2^-Tyr^3^-Thr^4^-Glu^5^-Ala^6^-Pro^7^-Gln^8^-Tyr^9^-Ile^10^ peptide and a C_15_ *β*-hydroxy fatty acid chain. b Linear fengycin A precusors ion [M+H]^+^ at *m/z* 1,481 containing a C_16_ *β*-hydroxy fatty acid chain.

**Relative Abundance (%)**

**600**

**800**

**1000**

**1200**

**1400**

**1600**

**1800**

**2000**

**m/z**

**0**

**10**

**20**

**30**

**40**

**50**

**60**

**70**

**80**

**90**

**100**

**995.49**

Supplementary Fig.S10 LC-ESI-MS/MS spectrum of the linear fengycin B precussors [M+H]^+^ at *m/z* 1,509 containing a C_16_ *β*-hydroxy fatty acid chain.
